# Subcortical brain structure in children with developmental coordination disorder: A T1-weighted volumetric study

**DOI:** 10.1007/s11682-021-00502-y

**Published:** 2021-08-13

**Authors:** Melody N. Grohs, Catherine Lebel, Helen L. Carlson, Brandon T. Craig, Deborah Dewey

**Affiliations:** 1 Department of Neurosciences, University of Calgary, Calgary, Canada; 2 Alberta Children’s Hospital Research Institute (ACHRI), Calgary, Canada; 3 Department of Radiology, University of Calgary, Calgary, Canada; 4 Hotchkiss Brain Institute (HBI), University of Calgary, Calgary, Canada; 5 Department of Pediatrics, University of Calgary, Calgary, Canada; 6 Department of Community Health Sciences, University of Calgary, Calgary, Canada; 7 Child Development Center, #397 Owerko Center, 2500 University Dr. NW, Calgary, AB T2N 1N4 Canada

**Keywords:** Neuroimaging, Subcortical, Structural, Developmental coordination disorder, Neurodevelopment

## Abstract

Developmental coordination disorder (DCD) is a neurodevelopmental disorder occurring in 5–6% of school-aged children. Converging evidence suggests that dysfunction within cortico-striatal and cortico-cerebellar networks may contribute to motor deficits in DCD, yet limited research has examined the brain morphology of these regions. Using T1-weighted magnetic resonance imaging the current study investigated cortical and subcortical volumes in 37 children with DCD, aged 8 to 12 years, and 48 controls of a similar age. Regional brain volumes of the thalamus, basal ganglia, cerebellum and primary motor and sensory cortices were extracted using the FreeSurfer recon-all pipeline and compared between groups. Reduced volumes within both the left and right pallidum (Left: F = 4.43, *p* = 0.039; Right: F = 5.24, *p* = 0.025) were observed in children with DCD; however, these results did not withstand correction for multiple comparisons. These findings provide preliminary evidence of altered subcortical brain structure in DCD. Future studies that examine the morphology of these subcortical regions are highly encouraged in order replicate these findings.

**Supplementary Information:**

The online version contains supplementary material available at 10.1007/s11682-021-00502-y.

## Introduction

Developmental coordination disorder (DCD) is a neurodevelopmental disorder characterized by motor impairment, which negatively impacts activities of daily living (American Psychiatric Association, 2013) as well as academic and psychosocial outcomes (Cairney, 2015). Motor deficits are evident from an early age and manifest as slow, inaccurate and/or clumsy movements (Wilson et al., 2013). Despite being a prevalent neurodevelopmental disorder, occurring in up to 6% of children (American Psychiatric Association, 2013), the aetiology of DCD remains unknown (Gomez & Sirigu, 2015).

Much of our current understanding of the neural correlates of DCD comes from studies that have employed functional magnetic resonance imaging (fMRI) and diffusion tensor imaging (DTI). FMRI studies report activity and functional connectivity differences among children with DCD compared to healthy controls (Biotteau et al., 2016); these differences have been reported within primary sensorimotor areas of the parietal and frontal lobes (Debrabant et al., 2013; Kashiwagi et al., 2009; Licari et al., 2015; McLeod et al., 2014, 2016; Querne et al., 2008; Reynolds et al., 2015; Zwicker et al., 2010, 2011), thalamic and basal ganglia structures (i.e., putamen, caudate, pallidum) (McLeod et al., 2014, 2016; Querne et al., 2008) and the cerebellum (Debrabant et al., 2013; McLeod et al., 2016; Zwicker et al., 2011). Similarly, DTI studies have found reduced anisotropy and diffusivity in cortical motor, thalamic and cerebellar pathways (Brown-Lum et al., 2020; Debrabant et al., 2016; Langevin et al., 2014; Zwicker et al., 2012). Given the converging evidence from fMRI and DTI studies, it has been hypothesized that the motor deficits observed in children with DCD may be related to dysfunction within cortico-striatal and cortico-cerebellar networks (Biotteau et al., 2016; Dewey & Bernier, 2016).

Despite the common notion of thalamic, basal ganglia and cerebellar involvement in DCD, few studies have investigated the macrostructure (i.e., volume) of these regions. Studies utilizing structural MRI in children with DCD have focused explicitly on cortical brain regions. One study, using voxel-based morphometry, found reduced premotor and frontal cortical volumes (Reynolds et al., 2017). Another study found thinner cortex in the frontal, parietal and temporal lobes (Langevin et al., 2015). Additionally, as DCD co-occurs with attention deficit hyperactivity disorder (ADHD) in up to 50% of cases (Dewey, 2018; Goulardins et al., 2015), Langevin and colleagues examined cortical thickness in children with co-occurring DCD and attention deficits and reported greater and more widespread reductions in cortical thickness among children with both DCD and ADHD (Langevin et al., 2015).

Shaw et al. (2016) and Dewey et al. (2019) provide preliminary evidence of reduced volumes within striatal and cerebellar regions in children who they defined as at risk for DCD, based on a parent questionnaire that asked about children’s motor skills on common daily tasks (Shaw et al., 2016) or low performance scores on a standardized motor exam (Dewey et al., 2019). However, children in these two studies were not clinically diagnosed and screening criteria was not comprehensive or in accordance with the criteria outlined in the Diagnostic and Statistical Manual of Mental Disorders (DSM-5) (American Psychiatric Association, 2013). It remains unclear if structural differences, such as altered volumes in thalamic, basal ganglia and cerebellar regions, are present in children who meet diagnostic criteria for DCD.

The cerebellum is involved in coordination, timing and planning of movements (Glickstein & Doron, 2008; Manto et al., 2012). The thalamus and basal ganglia play critical roles in movement planning, motor control and motor learning (Herrero et al., 2002; Lanciego et al., 2012). Given that children with DCD show deficits in these motor domains (Blank et al., 2019), there is a pressing need to understand the morphology of these brain regions. The current study used T1-weighted neuroimaging to investigate cortical (primary and secondary sensorimotor areas), thalamic, basal ganglia (caudate, putamen, pallidum) and cerebellar volumes in children with DCD. Based on previous findings of cortical thinning and reduced gray matter volumes, we hypothesized that children with DCD would display smaller volumes within the examined regions.

## Methods

### Participants

The current study combined two cohorts of children with DCD, aged 8 to 12 years. The first cohort included 19 children with DCD (mean age 9.7 $\pm$ 1.3 years; range 7.3–12.1 years; 9 (47%) male) and 19 controls (mean age 10.4 $\pm$ 1.3 years; range 8.6–12.6 years; 12 (63%) male), recruited between May 2012-August 2012 and scanned at the Seaman Family MR Research Center in Calgary, Alberta (Langevin et al., 2014). The second cohort included 18 children with DCD (mean age 10.2 $\pm$ 1.3 years; range 8.0–13.0 years; 12 (67%) male) recruited between July 2018-October 2019 and scanned at the Alberta Children’s Hospital in Calgary, Alberta. Controls within the same age range (n = 29; mean age 10.1 $\pm$ 1.7 years; range 7.4–13.0 years; 15 (52%) male) were selected from a separate study on typical brain and behavior development that used the same protocol and scanner at the Alberta Children’s Hospital (Andre et al., 2019). We refer to these cohorts as DCD1 and controls1 (cohort scanned at the Seaman Center) and DCD2 and controls2 (cohorts scanned at the Alberta Children’s Hospital), respectively.

Participants were recruited through developmental/community pediatricians, psychologists and physical/occupational therapists in Calgary, Alberta, as well as through advertisements on social media. Exclusion criteria for all cohorts were: (1) preterm birth (< 36 weeks’ gestation), (2) contraindications to magnetic resonance imaging and (3) a neuropsychiatric (other than ADHD), neurological and/or chronic disorder.

Children were classified as DCD if the four diagnostic criteria outlined in the DSM-5 were confirmed (American Psychiatric Association, 2013). Specifically, children demonstrated motor deficits (criterion A) based on Total Test scores below the 16^th^ percentile on the Movement Assessment Battery for Children-Second Edition (MABC-2). Motor deficits interfered with children’s daily functioning (criterion B), began early in development (criterion C) and were not better explained by an intellectual disability, visual impairment or neurological condition (criterion D); as confirmed by a parent questionnaire designed in-house, which included a detailed medical history of the child (see supplementary material), as well as the child demonstrating typical cognitive performance (Full-Scale IQ scores $\ge$ 80) on the Wechsler Abbreviated Scale of Intelligence-Second Edition (WASI-II). Given the high co-occurrence of ADHD with DCD (Dewey, 2018), children with ADHD were included in the DCD group (2 children in the DCD1 group and 10 in the DCD2 group). Diagnosis of ADHD by a registered physician was screened for using the in-house parent questionnaire outlined above. Children who were reported on the in-house parent questionnaire to be diagnosed with another developmental disorder such as autism spectrum disorder, Asperger syndrome or pervasive developmental disorder were excluded.

Children in the control group displayed typical motor development, confirmed by an MABC-2 Total Test scores above the 25^th^ percentile (controls1) or parent report on the in-house questionnaire, which included a detailed medical history of the child and specific questions regarding motor development (controls2), as well as typical cognitive performance on the WASI-II. Given the primary focus of this study was to investigate the structural correlates of motor deficits in children with DCD, a diagnosis of ADHD by a registered physician was not an exclusion criterion for controls (4 children in the controls1 group and none in the controls2 group had a confirmed diagnosis of ADHD based on parent report on the in-house questionnaire). Similar to the DCD groups, children identified with other developmental disorders on the parent questionnaire were excluded.

Written informed consent from participants’ legal guardians and child assent were obtained at enrollment. The University of Calgary Conjoint Health Research Ethics Board approved this research (REB18-0183; REB13-1346; REB15-1090).

### Motor and cognitive screening

The MABC-2 (Henderson et al., 2007) is a valid standardized motor assessment that evaluates motor performance across three domains: manual dexterity, aiming and catching and balance skills (Schoemaker et al., 2012; Van Waelvelde et al., 2007). The WASI-II is a short standardized assessment that provides a valid and reliable (reliability of 0.90) measure of intelligence (McCrimmon & Smith, 2013). Participants completed all four WASI-II subtests (Block Design, Vocabulary, Matrix Reasoning and Similarities).

### Magnetic Resonance Imaging (MRI) acquisition

MR imaging for the DCD1 and controls1 groups was performed at the Seaman Family MR Research Center in Calgary, Alberta, on a 3 Tesla General Electric (GE) Signa scanner with a 12-channel head coil (GE Healthcare, Milwaukee, WI, USA). A T1‐weighted spoiled gradient echo pulse sequence was acquired at rest (flip angle = 13°, repetition time = 7.4 ms, echo time = 3.1 ms, field of view = 256 mm, matrix = 256 × 256 pixels, slice thickness 0.8 mm, isotropic).

MR imaging for DCD2 and controls2 groups took place at the Alberta Children’s Hospital, Calgary, Alberta, on a GE 3 Tesla MR750w research system, equipped with a 32-channel head coil and 70 cm wide bore (GE, Waukesha, WI). T1-weighted images were acquired at rest (flip angle = 10°, repetition time = 8.2 ms, echo time = 3.2 ms, field of view = 256, matrix = 512 × 512, slice thickness 0.8 mm, isotropic).

### Magnetic Resonance Imaging (MRI) processing

Scans were quality checked by an investigator (MNG) blinded to participant group and demographics prior to pre-processing, to determine if they were of good quality or low-quality; 4 participants from the DCD1 group, 1 from controls1, 6 from DCD2 and 1 from controls2 were removed due to low quality scans, leaving the final number of participants as follows: DCD1 n = 19, controls1 n = 19, DCD2 n = 18, controls2 n = 29. Automated pre-processing and segmentation of T1-weighted anatomical scans were then conducted using FreeSurfer, V6.0.0 (Fischl, 2012). Briefly, the automated recon-all pipeline, with default settings, was used to perform: 1) skull stripping and brain extraction, 2) corrections for motion, head shape and position, 3) Talairach transformations, 4) intensity normalization, 5) segmentation of subcortical white and gray matter, 6) smoothing, topology correction and surface deformation, and 7) cortical and subcortical parcellation. This automated pre-processing approach has been described in more detail elsewhere (Dale et al., 1999). Following pre-processing, outputs were quality checked for skull stripping, segmentation and surface reconstruction errors by the same investigator (MNG). Manual corrections were performed if delineation of the pial surface and white matter boundary was poor, with defects spanning multiple sections within slices and/or consecutive slices. Placement of control points onto white matter voxels was done, followed by the recon-all -autorecon2 -cp processing command following the recommendations of FreeSurfer developers. (http://surfer.nmr.mgh.harvard.edu/fswiki/FsTutorial). All scans underwent 0–2 rounds of editing (0 rounds: 17 scans; 1 round: 51 scans; 2 rounds: 17 scans) and were quality checked post-processing to ensure boundary defects were corrected.

Estimates of total brain volume, as well as volumes for the thalamus, basal ganglia structures (i.e., caudate, putamen, pallidum), cerebellum, and pre-central (primary motor cortex), post-central (primary sensory cortex) and para-central regions were extracted. Volumes were extracted separately for the left and right hemispheres.

### Statistical analysis

Statistical analysis was performed in Jamovi (Şahin & Aybek, 2019, V1.8.1) and SPSS (IBM SPSS Software, V25.0). Age and sex were compared between the DCD (DCD1, DCD2) and control groups (controls1, controls2) using an independent samples t-test or chi-square test, respectively.

A linear mixed effects model was chosen for the primary analysis, which examine regional brain volume differences between controls (controls1, controls2) and the DCD groups (DCD1, DCD2), controlling for total brain volume, with fixed effects for Group and random effects for Scanner. Total brain volume was controlled for given that significant changes in brain volumes continue to occur between the ages 8–12 years (Giedd et al., 1999; Lenroot & Giedd, 2006). Given the use of two different scanners, this statistical approach allowed for the data to be treated as a two-level structure with participants nested within scanner sites. We report results uncorrected and corrected for multiple comparisons using Benjamini–Hochberg False Discovery Rate (FDR) (18 multiple comparisons were performed with an FDR of 0.05).

As 4 controls had a diagnosis of ADHD, two secondary analyses were conducted: 1) ADHD was included as a covariate, and 2) the controls with ADHD were removed. Finally, linear regressions were used to investigate relationships between motor performance and regional brain volumes; specifically, the relationships between MABC-2 Total Test standard scores and the volumes of regions showing significant between group differences, controlling for scanner and total brain volume. It is important to note that controls2 group was not assessed using the MABC-2; therefore, these regressions included only participants with a Total Test score on the MABC-2 (DCD1, controls1, DCD2).

## Results

### Participants

Group demographics and clinical characteristics are shown in Table 1. No group differences were observed for age (t(83) = 0.839, *p* = 0.404) or sex ($\chi$ ^2^(1) = 0.002, *p* = 0.963).

**Table 1:** Participant demographics and clinical characteristics. Test statistics are reported for group differences between DCD participants (DCD1 & DCD2) and controls (controls1 & controls2)

	DCD1	Controls1	DCD2	Controls2	Test Statistic	p value
N	19	19	18	29	-	-
Sex (% Male)	47	63	67	52	χ^2^(1) = 0.002	0.963
Age (mean years ± SD)	9.7 ± 1.3	10.5 ± 1.3	10.2 ± 1.3	10.1 ± 1.7	t(83) = 0.839	0.404
MABC-2 Total Score (mean standard Score ± SD)	5.53 ± 1.68	9.58 ± 1.35	2.84 ± 1.68	-	-	-
WASI-II Full-Scale IQ (mean ± SD)	104 ± 20	109 ± 14	103 ± 13	109 ± 12	t(83) = 1.537	0.128
Co-occurring Attention Deficit (n(%))	2(11)	4(21)	10(55)	0(0)	χ^2^(1) = 6.079	0.014

### Clinical characteristics

As per inclusion criteria, all children in the DCD1 and DCD2 groups scored below the 16^th^ percentile on the MABC-2 (mean Total Test standard score DCD1: 5.53 ± 1.68; DCD2: 2.84 ± 1.68) and children from the controls1 group scored above the 25^th^ percentile (mean Total Test standard score 9.58 ± 1.35). None of the children in the controls2 group had been diagnosed with a motor disorder based on the in-house parent questionnaire; they were, however, not formally assessed using the MABC-2. Typical cognitive performance was demonstrated by all children.

### Group volumetric differences

**Fig. 1:**
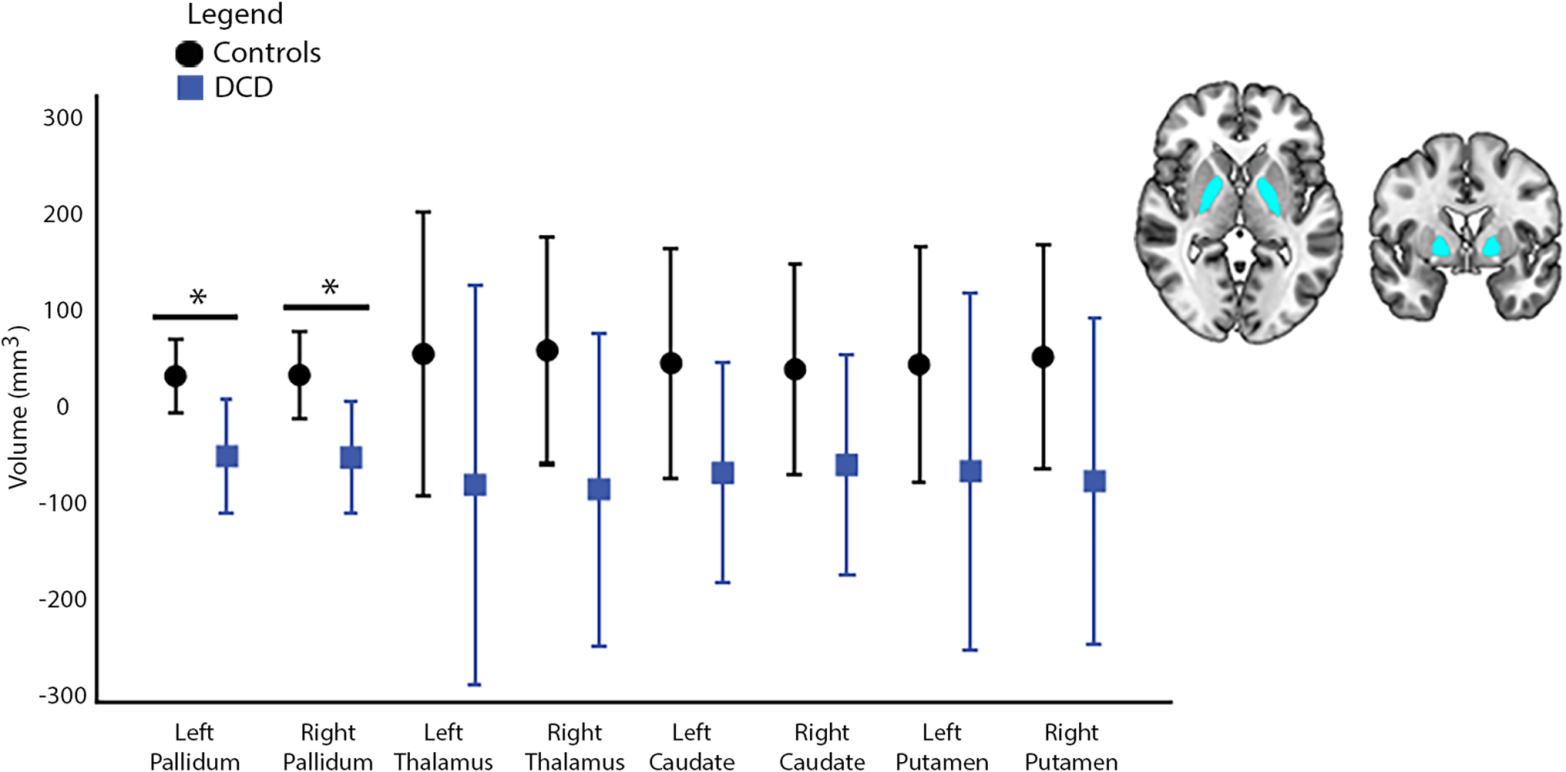
Group comparisons for subcortical volumes. Volumes for the DCD (blue bars) and control (black bars) groups are shown as residuals, regressing out total brain volume and scanner effects. Error bars represent 95% confidence intervals. Brain image shows significant regions of interest. *Indicates significant results, uncorrected for multiple comparison corrections (*p* < 0.05)

**Fig. 2:**
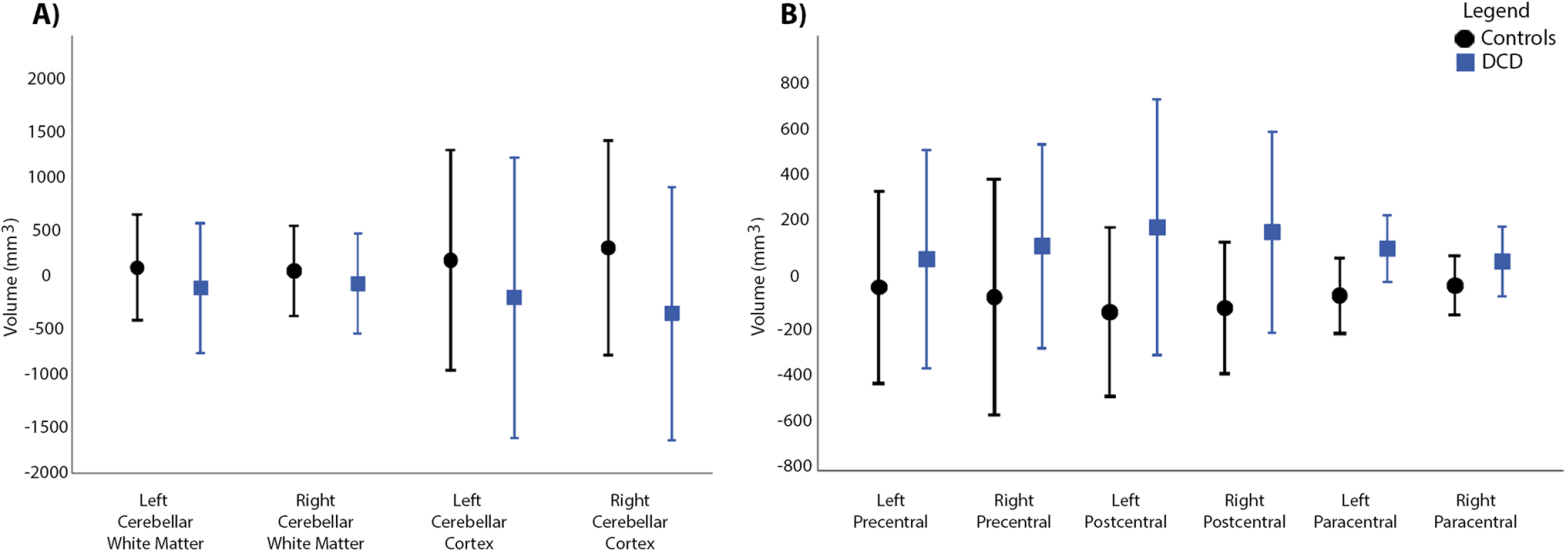
Group comparisons for cortical and cerebellar volumes. Volumes for the DCD (blue bar) and control (black bar) groups are shown as residuals, regressing out total brain volume and scanner effects. Error bars represent 95% confidence intervals. *Indicates significant results, uncorrected for multiple comparison corrections (*p* < 0.05)

Between group volumetric differences were observed for the left and right pallidum (L: F = 4.43, *p* = 0.039, 95% CI [-143.0, -5.13]; R: F = 5.24, *p* = 0.025, 95% CI [-165.0, -12.8]) (Fig. 1), such that, lower mean volumes in these regions were observed in children with DCD compared to the controls. Findings did not survive corrections for multiple comparisons. Reduced bilateral pallidal volumes in the DCD group remained significant when ADHD was included as a covariate (L: F = 7.31, *p* = 0.008, 95% CI [-166.0, -26.5]; R: F = 4.32, *p* = 0.041, 95% CI [-161.0, -4.77]) as well as when the 4 control participants with diagnosed ADHD were removed (L: F = 4.12, *p* = 0.044, 95% CI [-145.0, -2.52]; R: F = 4.22, *p* = 0.043, 95% CI [-145.0, -3.42]). No other group differences were noted in remaining cortical, subcortical or cerebellar volumes (Fig. 2; Supplementary Table 1).

### Structure–function correlates

Volumes of the left and right pallidum did not predict MABC-2 Total Test standard scores (Table 2).

**Table 2:** Results of linear regressions examining the associations between MABC-2 Total Test standard scores and volumes of the right and left pallidum. Results are shown for the control and DCD groups combined, controlling for scanner and total brain volume, as well as for Control and DCD groups separately, controlling for total brain volume. LCI: lower level 95% confidence interval, UCI: upper level 95% confidence interval

	MABC-2 Total Test Standard Score		
	All Participants (n = 56) *p* (LCI, UCI)	Control Participants (n = 19) *p* (LCI, UCI)	DCD Participants (n = 37) *p* (LCI, UCI)
Right Pallidum	0.804 (-0.232, 0.298)	0.462 (-0.654, 0.311)	0.512 (-0.155, 0.305)
Left Pallidum	0.859 (-0.245, 0.205)	0.751 (-0.473, 0.348)	0.534 (-0.140, 0.266)

## Discussion

Converging evidence suggests that dysfunction within cortico-striatal and cortico-cerebellar networks may contribute to the motor deficits seen in children with DCD (Biotteau et al., 2017; Dewey & Bernier, 2016). Yet, to date very limited research has examined brain morphology within thalamic, basal ganglia and cerebellar regions. Here, we show preliminary evidence of smaller brain volumes within the pallidum among a sample of children with DCD.

The pallidum is one of the major output structures of the basal ganglia and plays a fundamental role in motor control and movement selection (Grillner et al., 2005; Kretschmer, 2000; Nambu et al., 2002). Afferents from the cerebellum and structures of the basal ganglia, including the pallidum, project to motor or somatosensory cortices via different nuclei of the thalamus (Alexander et al., 1990; Haber & Calzavara, 2009). These motor pathways contribute to the production of motor movements in response to sensory stimuli and play a key role in motor control and learning (Sommer, 2003). Previous research has reported that greater pallidal volumes were associated with better motor scores in children (Bolk et al., 2018; Loh et. al., 2019). Additionally, reduced volumes within the pallidum have been reported in adults (van den Bogaard et al., 2011; Georgiou-Karistianis et al., 2013; Motl et al., 2015; Gooijers et al., 2016; Coppen et al., 2018) and children with motor impairment (Estes et al., 2011; Dewey et al., 2019). The current findings may suggest similar subcortical volume reductions in children with DCD and provides early support to the theory that focal differences in relevant brain regions may contribute to the motor difficulties observed in affected children.

Contrary to previous neuroimaging findings (Biotteau et al., 2016), we observed no significant group differences in brain volumes within cortical (pre-, post- and para-central regions) or cerebellar regions. This discrepancy could be related to heterogeneity across studies in both design and populations. For instance, some studies investigated brain structure corrected for total brain volume (Reynolds et al., 2017) while others investigated uncorrected brain structure (Dewey et al., 2019; Langevin et al., 2015; Shaw et al., 2016). Using uncorrected values may make it difficult to determine if the observed macrostructural differences are a result of differences in brain size between participants or regional structure (Brain Development Cooperative Group, 2012).

With regards to population, different cutoff scores have been used to identify individuals with DCD. Some studies also included children with DCD who were born preterm or had co-occurring disorders, such as ADHD as in the present study. These numerous factors that vary across studies could be driving the differences noted in brain structure. For example, altered cortical and subcortical brain structure has previously been described in children born preterm (Dewey et al., 2019; Loh et al., 2017, 2019; Ment et al., 2009). It is therefore, vital that larger studies including similar samples and study designs are undertaken to try to replicate the current findings, before any definitive conclusions regarding the presence of brain structural differences in children with DCD can be made.

Numerous FMRI and DTI studies suggest altered cortico-striatal and cortico-cerebellar networks in children with DCD (Biotteau et al., 2016). However, given the limited structural differences observed in the current study, it is possible that altered macrostructural abnormalities, such as volume, within these regions may not be strongly associated with DCD. Furthermore, altered motor circuitry may not be detectable with coarser measures such as volume.

Despite preliminary findings of decreased subcortical volumes among children with DCD, we did not observe a relationship between regional volumes and MABC-2 standard scores. The absence of an association between brain structure and motor functioning could be because the MABC-2 is not sensitive or specific enough to capture potential structure–function relationships. Future studies that include tasks more directly related to the functional correlates of the basal ganglia (i.e., measures of motor control, motor learning and bilateral motor skills) (Doyon et al., 2009; Turner & Desmurget, 2010; Gooijers et al., 2016) may be more likely to reveal relationships.

Strengths of the current study include a considerably larger sample of children with DCD, as well as more comprehensive screening and demographic data than previous neuroimaging studies investigating brain macrostructure in DCD (Biotteau et al., 2016; Wilson et al., 2017). However, this study with its current sample of 37 children with DCD and 48 controls may still be underpowered as our significant findings related to the pallidum did not survive correction for multiple comparisons. Our study is also limited by its cross-sectional design. To help elucidate if structural brain differences in children with DCD are present early in life and if they persist or change, throughout childhood and into adulthood longitudinal research studies are needed (Dewey et al., 2019). Another limitation is our use of data from different scanners. Intensity differences can arise between scanners and subcortical brain regions intrinsically have poorer contrast and may be more susceptible to variability in contrast due to scanner differences (Stonnington et al., 2008). Therefore, the inclusion of images from two scanners could have limited our power to detect volumetric brain differences between groups. As it is challenging to recruit and scan large populations of children with DCD at one site, further research examining potential scanner effects in relation to brain volume differences is essential in order to support the conduct of larger multi-centred studies on this population.

It is also important to note that three of our cohorts included children with diagnosed ADHD (controls1, DCD1, DCD2). Previous studies have shown altered brain structure in children with ADHD (Samea et al., 2019), as well as more pronounced brain structural differences in children with co-occurring DCD and ADHD (Langevin et al., 2015). Considering previous findings, we included secondary analyses controlling for the presence of ADHD as well as removing controls with an ADHD diagnosis. Importantly, findings remained stable, suggesting that the brain structural differences observed here may be associated with DCD as opposed to ADHD. Furthermore, motor difficulties were excluded from our controls2 group via parent questionnaire; however, these children did not participate in a standardized motor assessment. Therefore, we cannot be certain if any of these participants had an undiagnosed motor difficulty. Future larger studies are required to verify our findings and to examine the effects of comorbidities on brain structure in children with DCD.

## Conclusions

The current study provides preliminary evidence of altered subcortical brain structure in children with DCD. Given the limited number of structural neuroimaging studies in children with DCD, as well as the mixed findings across these studies, further research to replicate findings is necessary. Defining a neural signature in DCD and linking the observed motor deficits to potential structural variants in localized brain regions could help inform future targeted interventions. Identifying different brain structural variants may also help to better understand the heterogeneity of the symptomology observed in DCD.

## Supplementary Information

Below is the link to the electronic supplementary material.

Supplementary file1 (DOCX 1586 KB)

## Data Availability

Data will be made available upon reasonable request.
