# Supplementary material for: Subcortical brain structure in children with developmental coordination disorder: A T1-weighted volumetric study
Source: Brain Imaging Behav. 2021 Aug 13;15(6):2756–65. doi: 10.1007/s11682-021-00502-y (PMC8761714; doi:10.1007/s11682-021-00502-y)

**Subcortical Brain Structure in Children with Developmental Coordination Disorder: A T1-Weighted Volumetric Study.**

Melody N. Grohs^1,2^, Catherine Lebel^2,3,4^, Helen L. Carlson ^2,4,5^, Brandon T. Craig^1,2,4^ & Deborah Dewey^2,4,5,6^

1 Department of Neurosciences, University of Calgary

2 Alberta Children’s Hospital Research Institute (ACHRI)

3 Department of Radiology, University of Calgary

4 Hotchkiss Brain Institute (HBI), University of Calgary

5 Department of Pediatrics, University of Calgary

6 Department of Community Health Sciences, University of Calgary

**Corresponding Author:**

Dr. Deborah Dewey

Ph: 1(403)441-8468

Em: [dmdewey@ucalgary.ca](mailto:dmdewey@ucalgary.ca)

#397 Owerko Center, Child Development Center, 2500 University Dr. NW, Calgary, AB T2N 1N4

**Supplementary Material:**

| **Region of Interest (Volume)** | **Group Comparison Statistics**  F *p* [LLCI, ULCI] | | |
| --- | --- | --- | --- |
| Left Cerebellum White Matter | 0.015 | *0.905* | [-824, 932] |
| Left Cerebellum Cortex | 0.150 | *0.699* | [-1459, 2179] |
| Right Cerebellum White Matter | 0.108 | *0.743* | [-597, 838] |
| Right Cerebellum Cortex | 0.020 | *0.887* | [-1553, 1796] |
| Left Thalamus | 0.661 | *0.419* | [-370, 153] |
| Left Caudate | 0.885 | *0.706* | [-251, 88.2] |
| Left Putamen | 2.580 | *0.113* | [-432, 43.0] |
| **Left Pallidum** | **4.430** | ***0.039**** | **[-143, -5.13]** |
| Right Thalamus | 1.660 | *0.202* | [-349, 72.4] |
| Right Caudate | 0.140 | *0.710* | [-193, 131] |
| Right Putamen | 2.950 | *0.096* | [-361, 23.9] |
| **Right Pallidum** | **5.240** | ***0.025**** | **[-165, -12.8]** |
| Left Paracentral | 3.800 | *0.055* | [-1.19, 446] |
| Left Postcentral | 0.189 | *0.706* | [-492, 773] |
| Left Precentral | 0.010 | *0.922* | [-562, 621] |
| Right Paracentral | 2.200 | *0.142* | [-47.3, 342] |
| Right Postcentral | 0.343 | *0.558* | [-358, 663] |
| Right Precentral | 1.640 | *0.204* | [-240, 1141] |

**Supplementary Table S1: Test statistics for group comparisons of regional brain volumes, controlling for scanner and total brain volume.** *Indicates significant results uncorrected for multiple comparisons (*p*<0.05).


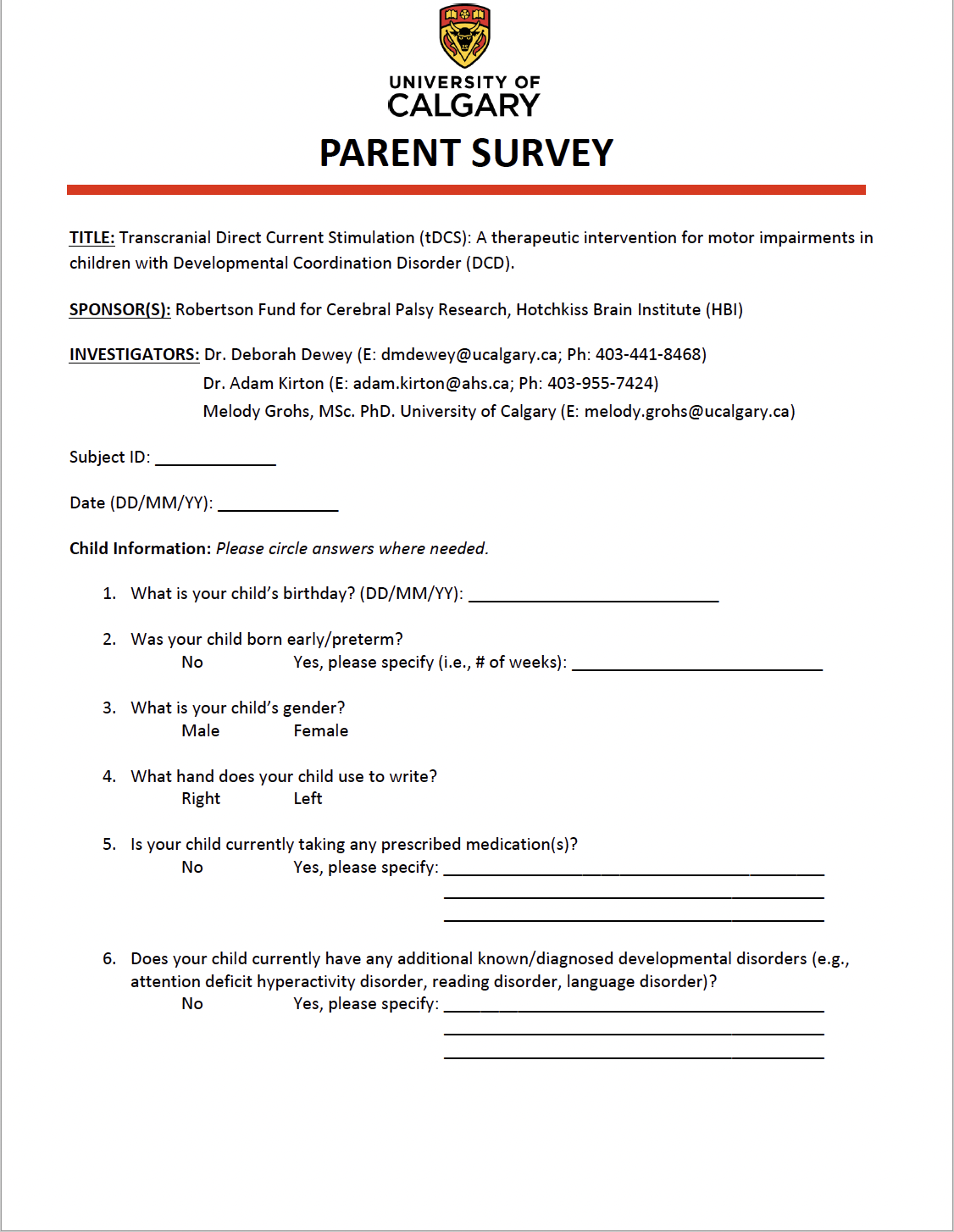


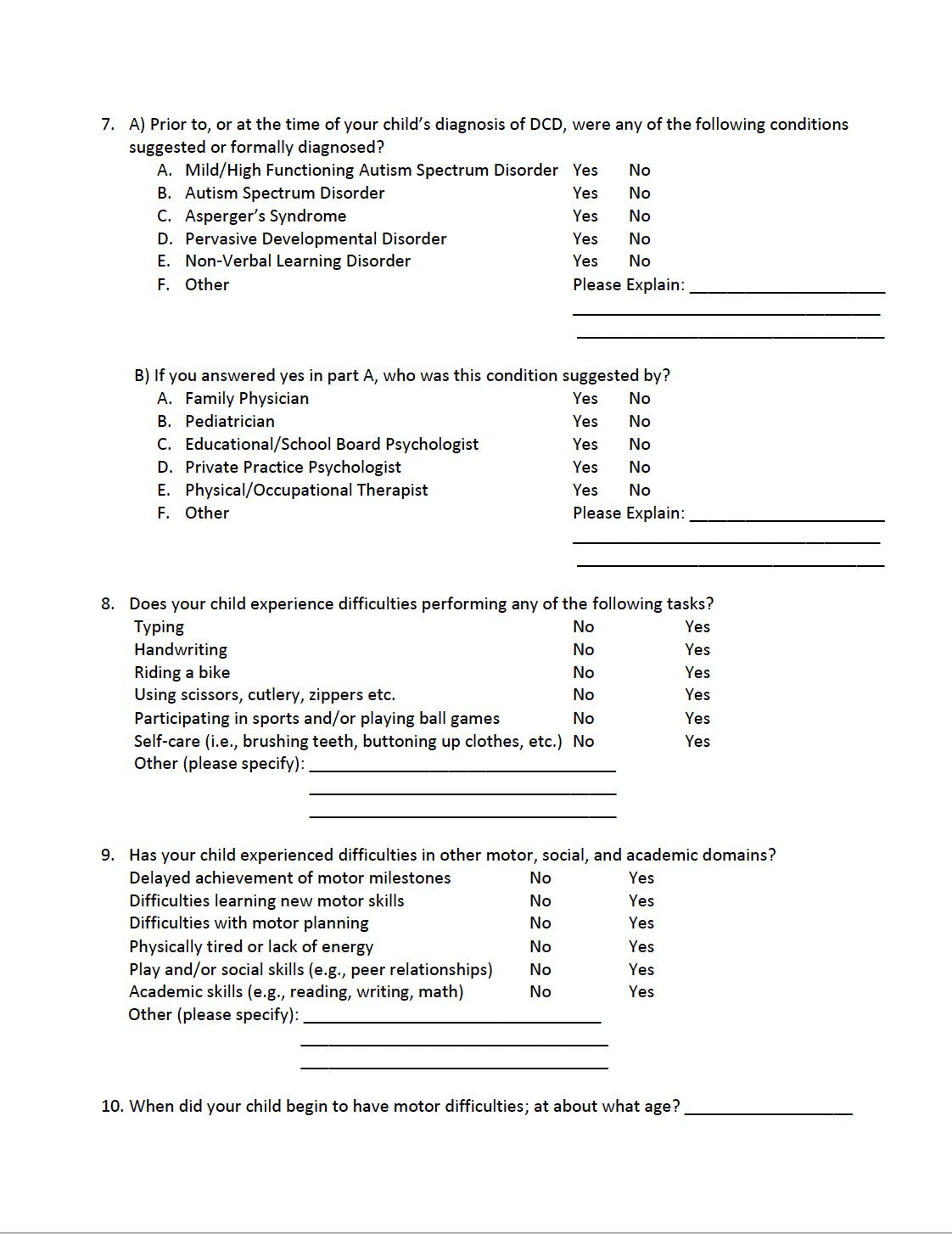


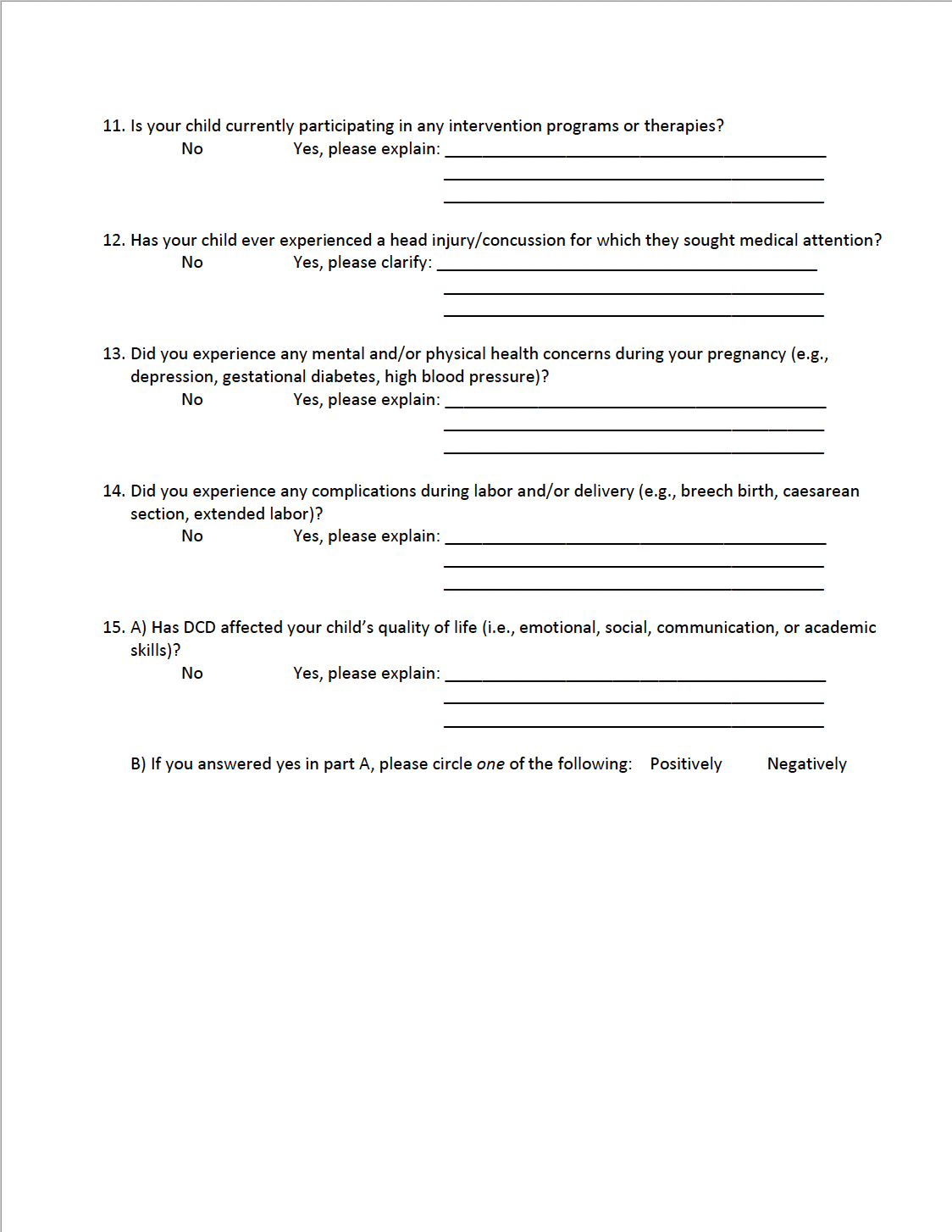

Supplement: Supplementary file 1 — Supplementary file1 (DOCX 1586 KB) [file 11682_2021_502_MOESM1_ESM.docx]
